# A Simplified Method for Simultaneous Determination of α-Lipoic Acid and Low-Molecular-Mass Thiols in Human Plasma

**DOI:** 10.3390/ijms21031049

**Published:** 2020-02-05

**Authors:** Kamila Borowczyk, Patrycja Olejarz, Grażyna Chwatko, Marcin Szylberg, Rafał Głowacki

**Affiliations:** 1 Department of Environmental Chemistry, Faculty of Chemistry, University of Lodz, Pomorska 163, 90-236 Łódź, Poland; patrycja.olejarz@o2.pl (P.O.); grazyna.chwatko@chemia.uni.lodz.pl (G.C.); rafal.glowacki@chemia.uni.lodz.pl (R.G.); 2 Rehabilitation Center “Kraszewski”, Kraszewskiego 7/9, 93-161 Łódź, Poland; szylberg_m@poczta.onet.pl

**Keywords:** α-lipoic acid, low-molecular-mass thiols, simultaneous reduction and derivatization, high performance liquid chromatography, ultraviolet detection

## Abstract

α-Lipoic acid, glutathione, cysteine, and cysteinylglycine can be applied as therapeutic agents in civilization diseases such as diabetes mellitus, cardiovascular diseases, and cancers. On the other hand, a higher concentration of homocysteine can result in health problems and has been indicated as an independent risk factor for cardiovascular disease and accelerated atherosclerosis. Here, the first simplified HPLC-UV assay that enables simultaneous determination of α-lipoic acid and low-molecular-mass thiols in plasma, reduces the number of steps, shortens the total time of sample preparation, and limits the amount of single-use polypropylene laboratory materials is described. The assay is based on reversed-phase high performance liquid chromatography with UV detection and simultaneous reduction of disulfide bound with *tris*(2-carboxyethyl)phosphine and the selective pre-column derivatization of the thiol group with 1-benzyl-2-chloropyridinium bromide. Linearity in the detector responses for plasma samples were observed in ranges: 0.12–5.0 nmol mL^−1^ for α-lipoic acid; 2.0–20.0 nmol mL^−1^ for glutathione, cysteinylglycine, and homocysteine; and 40.0–400.0 for cysteine. The LODs for α-lipoic acid and low-molecular-mass thiols were 0.08 and 0.12 nmol mL^−1^, respectively, while LOQs were 0.12 and 0.16 nmol mL^−1^, respectively. The usefulness of the proposed method has been proven by its application to real samples.

## 1. Introduction

**Figure 1:**
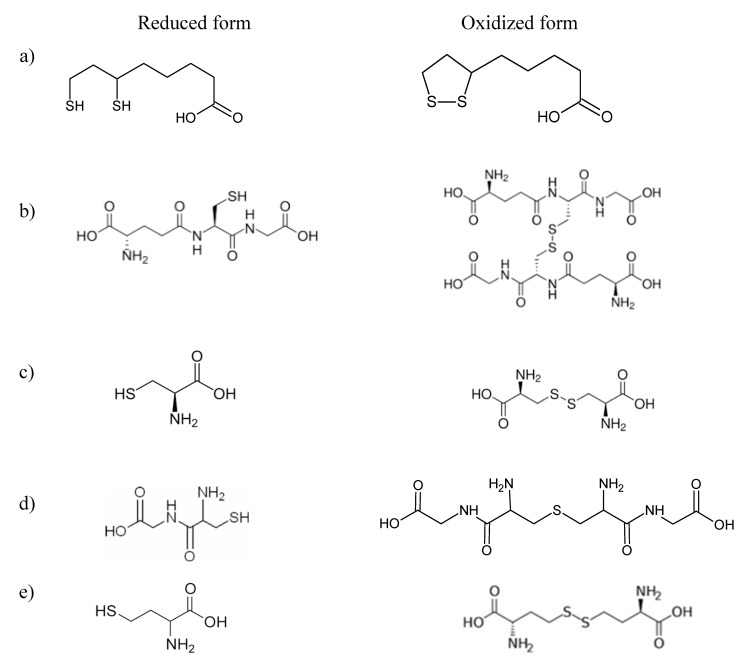
Chemical formulas of reduced and oxidative forms of some endogenous thiols: (**a**) dihydrolipoic acid and α-lipoic acid; (**b**) reduced glutathione and oxidized glutathione; (**c**) cysteine and cystine; (**d**) reduced cysteinylglycine and oxidized cysteinylglycine; (**e**) homocysteine and homocystine.

Thioctic acid, chemical name 1,2-dithiolane-3-pentanoic acid, is commonly known as α-lipoic acid (LA) (Figure 1a). In the human body, it is naturally synthesized in the liver and other tissues. It is also provided in the diet [1]. In humans, LA plays a key role as an essential co-factor for several mitochondrial multi-enzyme complexes involved in energy metabolism, such as the pyruvate dehydrogenase and α-ketoglutarate dehydrogenase complexes. LA possesses the ability to scavenge oxygen species and to regenerate other antioxidants [2]. Although, in mitochondria, LA is reduced to its thiol form, dihydrolipoic acid (DHLA), the powerful antioxidant properties are retained in both forms [3]. Some randomized clinical trials have proven that LA can be applied as a therapeutic agent in civilization diseases such as diabetes mellitus [4], cardiovascular diseases [5], and cancers [6]. The mechanism of work of LA is based on improving glucose-insulin homeostasis and decreasing chronic inflammation [1]. It has been proven that LA exhibits anti-tumor activities in several cancer models by impacting several hallmarks on most of the signaling pathways implicated in proliferation, invasion, migration, epithelial–mesenchymal transition, stemness and apoptosis [2]. Very recent pre-clinical and limited clinical trial evidence have suggested LA as a leading candidate in multiple sclerosis therapy [7]. This compound is able to regulate the immune system in either direct or indirect ways. Studies reviewed in 2019 suggest that LA can be used to treat autoimmune diseases, including systemic lupus erythematosus, rheumatoid arthritis, and primary vasculitis [8]. On top of this, LA reduces the progression of cellular degeneration and improves retinal function [9].

Similarly to LA, glutathione (GSH) is a non-enzymatic, endogenous, direct antioxidant. Both of them are typical, small molecule scavengers that bind reactive oxygen species. GSH (Figure 1b) is the most prevalent antioxidant in the brain, found in millimolar concentrations in most cells. Reduced GSH reacts with free radicals to form oxidized glutathione (GSSG); this form can occur independently or it can be catalyzed by the enzyme, glutathione peroxidase. In the next step, GSSG is recycled back to two GSH molecules by GSH reductase using the reducing equivalents of nicotinamide adenine dinucleotide phosphate [10]. GSH levels are decreased in diseases based on oxidative stress, including Alzheimer’s disease and aging [11]. It has been found that with an increasing progression of Alzheimer’s disease, GSSG and GSSG/GSH levels also increase. Additionally, a linear correlation between increased GSSG levels and the decreased cognitive status of patients suffering from Alzheimer’s disease was observed [8]. Another finding suggests that there may be GSH deficits and abnormalities in the GSH redox cycle in patients with schizophrenia [12]. GSH is also involved in several metabolic processes, such as synthesis of proteins and DNA, enzyme activity, metabolism, gene expression, signal transduction, and the intensification of cytoplasmic and transmembrane transport [13]. GSH is involved in several pathological pathways and plays an important role in cancer and regulation of the progression through the cell cycle, cell survival, growth, and death [14]. It must be highlighted that decreasing GSH levels and related enzymes in cancer cells may be a therapeutic target for cancer treatment [15].

GSH is comprised of three amino acids, glutamate, cysteine (Cys), and glycine. Glutamate and glycine are found in millimolar concentrations, whereas free Cys (Figure 1c) is limited, with most non-protein Cys being stored within GSH. Because the physiological amount of brain-resident Cys and cysteinylglycine (Cys–Gly) (Figure 1d) limits the formation of GSH, most current research have focused on increasing Cys levels in the brain as an indirect way to increase GSH levels [10]. The concentration of Cys in the human body is determined by the level of N-acetyl-cysteine and also by the process called transsulfuration, in which homocysteine (Hcy) formed from the dietary methionine is transferred to Cys. The first step of transsulfuration is catalyzed by cystathionine β-synthase [16,17]. Hcy (Figure 1e), a type of amino acid that is naturally found in blood, is not harmful at normal levels. Elevated levels of this amino acid called hyperhomocysteinemia can result in health problems and have been indicated as an independent risk factor for cardiovascular disease and accelerated atherosclerosis [18,19]. Recent results suggest that the increased Hcy level is positively correlated with low-density lipoprotein cholesterol (LDL-C) levels in hypothyroidism patients. A potential harmful correlation may exist between Hcy and LDL-C under the condition of hypothyroidism [20]. To clarify functions of LA, GSH, Cys, Hcy and Cys–Gly in biochemical and clinical practice, the control all of these in the human body is required.

The high separation capacity of high performance liquid chromatography (HPLC) makes it the preferred technique for biological samples analysis. For quantification of LA in biological fluids, mainly in human plasma, several HPLC methods which exploit spectrophotometric [21], spectrofluorometric [22,23,24], electrochemical [25,26], and mass spectrometry [27] detection have been developed and described in the literature. Although these assays allow quantification of LA in human plasma, they do not give the possibility to control levels of metabolically important endogenous amino thiols such as GSH, Cys, Hcy, and Cys-Gly. Assays based on UV detection and HPLC or capillary electrophoresis analysis dedicated to low-molecular-mass thiols detection and determination have been summarized by Bald and co-authors [28].

The biological fluid most commonly analyzed is plasma [28,29]. Human serum albumin (HSA) is the most abundant plasma protein and accounts for 50% of the total plasma proteins. From the analytical point of view, HSA and other proteins present in plasma can modify the chromatographic column and preclude separation. To avoid these troubles, usually deproteinization with the use of trichloroacetic acid (TCA) or organic solvents such as methanol, acetonitrile, acetone, and centrifugation, followed by removal of the protein pellet are required [28,29]. The other possibility to dodge the problem of chromatographic column modification is an application of chromatographic columns dedicated to protein analysis [30].

Here we describe the first simplified HPLC-UV assay that offers a simultaneous determination of LA and low-molecular-mass thiols in human plasma, reduces the number of steps, shortens the total time of sample preparation and limits the amount of single-use polypropylene laboratory materials, such as tips and tubes.

## 2. Results and Discussion

LA and sulfur-containing amino acids and peptides, such as Cys, Hcy, GSH, and Cys–Gly, play important roles in human health. It indicates the need to control the concentration of each of these compounds. Although many analytical protocols dedicated to the determination of LA or metabolically related amino thiols in human plasma have been presented in the literature, all of them require the determination of LA and GSH, Cys, Hcy in two different assays [21,22,23,24,25,26,27,28,29,30].

Taking under consideration protocols described in previously published papers, the simultaneous determination of antioxidants such as LA and GSH, and its metabolic relatives in human plasma samples seems to be challenging. The assays require four steps of sample preparation protocols, such as reduction of disulfide bonds, derivatization of the thiol group, deproteinization and centrifugation for protein removal [21,22,23,24,25,26,27,28,29,30]. All of these compounds occur in the human body mainly in the oxidative form not accessible for the derivatization reagent. For this reason, the reduction of the disulfide bond is obligatory. On the other hand, thiols are highly polar and water-soluble, which makes their extraction from biological matrices almost impossible without chemical derivatization. Moreover, thiols lack the structural properties necessary for commonly used chromatographic detectors such as UV–Vis absorbance and fluorescence [28,29,30]. Therefore, the analyst must harness to derivatization for signal enhancement and protection of sulfhydryl group. The last step of the analytical protocol is deproteinization. It is important due to the fact that proteins in plasma samples present a variety problems: among others, a large number of individual compounds, difficulty in resolving the analytes of interest, low concentrations of exogenous or endogenous compounds of interest, and the presence of proteins can modify the chromatographic column and preclude separation [28,29,30]. The separation of protein pellet from supernatant is an additional step based on the transfer of supernatant. Thus, the deproteinization and centrifugation extend the time of sample preparation. The second disadvantage of deproteinization is the fact that LA as an amphiphilic molecule, possesses both hydrophilic and hydrophobic fragments and can specifically interact with the surface of proteins. It was proven that acidic deproteinization of plasma proteins markedly adsorb LA [21]; consequently, the analyte can be accidentally removed from a sample with precipitated proteins and concentration of LA in the solution becomes lower than expected. To counteract this problem, an addition of MeCN is recommended; however, this step causes additional sample dissolving [21]. Another drawback of deproteinization is increasing the number of polypropylene tubes using during sample preparation. This problem must be stressed in ”the age of plastic”. All of these aspects were considered during the elaboration of the new method for the simultaneous determination of LA and low-molecular-mass amino thiols in human plasma.

### 2.1. The Thiol Group Derivatization

**Figure 2:**
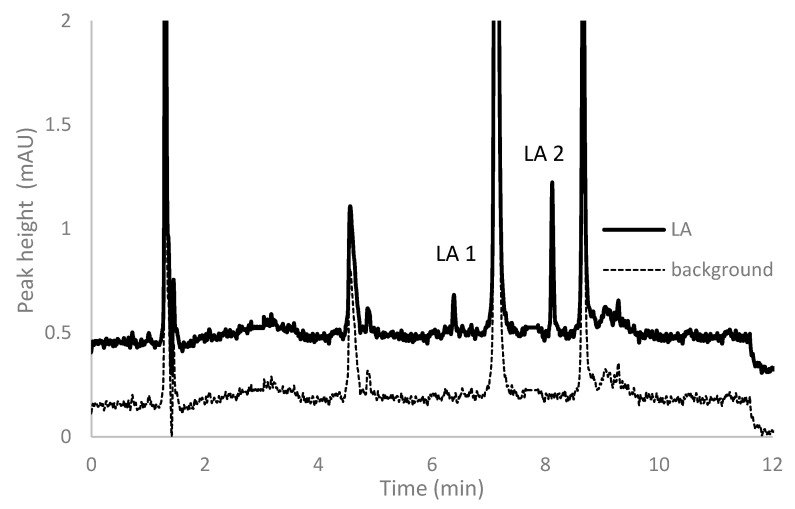
Chromatogram of α-lipoic acid after pre-column simultaneous reduction with *tris*(2-carboxyethyl)phosphine and derivatization with 1-benzyl-2-chloropyridinium bromide in low excess of the derivative reagent.

Compounds with activated halides, such as 2-chloro-1-methyllepidinium tetrafluoroborate (CMLT), 2-chloro-1-methylquinolinium tetrafluoroborate (CMQT), 1-benzyl-2-chloropyridinium bromide (BCPB), 2-chloro-1-methylpyridinium iodide (CMPI), and 2-chloro-1-propylpyridinium iodide (CPPI) are commonly used derivatization reagents dedicated to determination of endogenous and exogenous thiols in various real-world samples [14,28,30,31,32,33,34] with the use of the HPLC–UV technique. On the other hand, for the derivatization of LA in plasma samples, mainly BCPB is recommended so far [21,34,35]. During the method development, we tested three of the most commonly used derivatization reagent—CMLT, CMQT, and BCPB. In all cases, we found that after LA derivatization, two different signals were observed (Figure 2). It was explainable due to the fact that LA in its reduced form, DHLA, possesses two thiol groups that can react with the derivatizing reagent. It is commonly known that the proper conditions of the derivatization reaction, such as temperature, pH, excess of the compound used for derivatization, affect the final structure of the final derivative [28,29,30].

**Figure 3:**
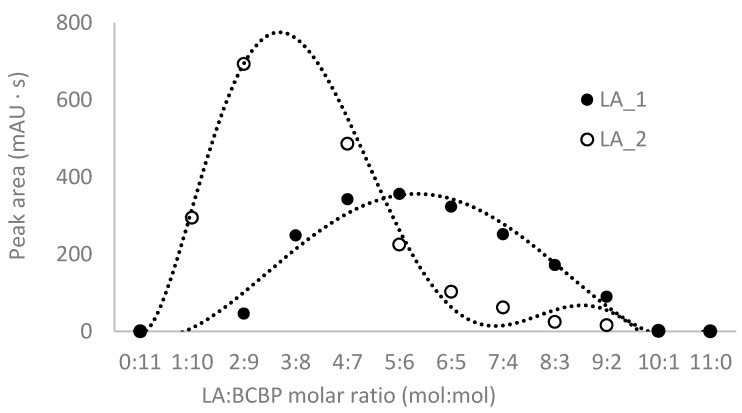
Estimation of stoichiometric molar ratio by continuous variation method for the reaction of α-lipoic acid with 1-benzyl-2-chloropyridinium bromide. The reaction was carried out in 0.2 mol·L^−1^, phosphate buffer, pH = 9.

**Figure 4:**
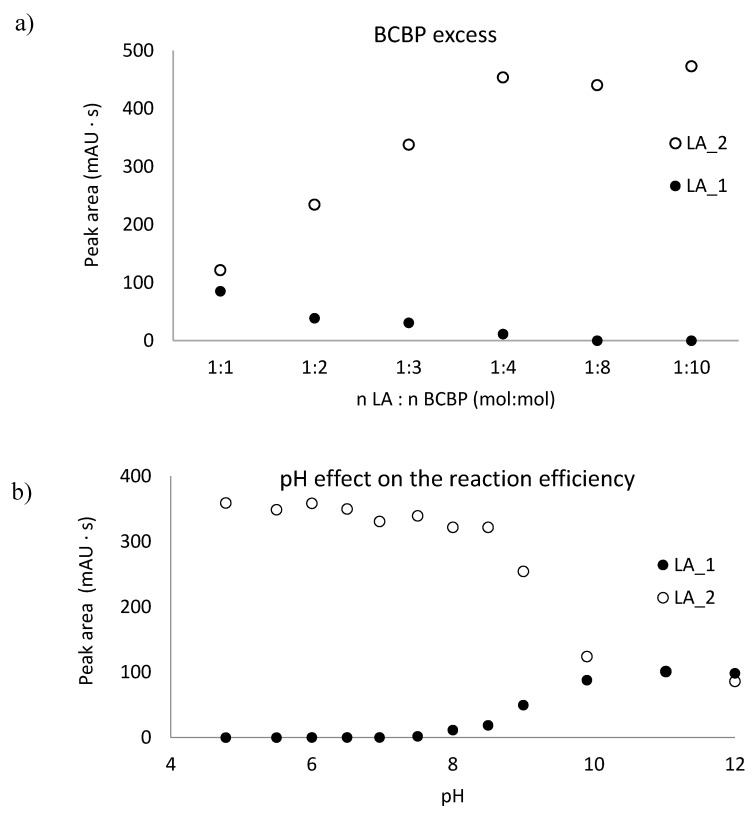

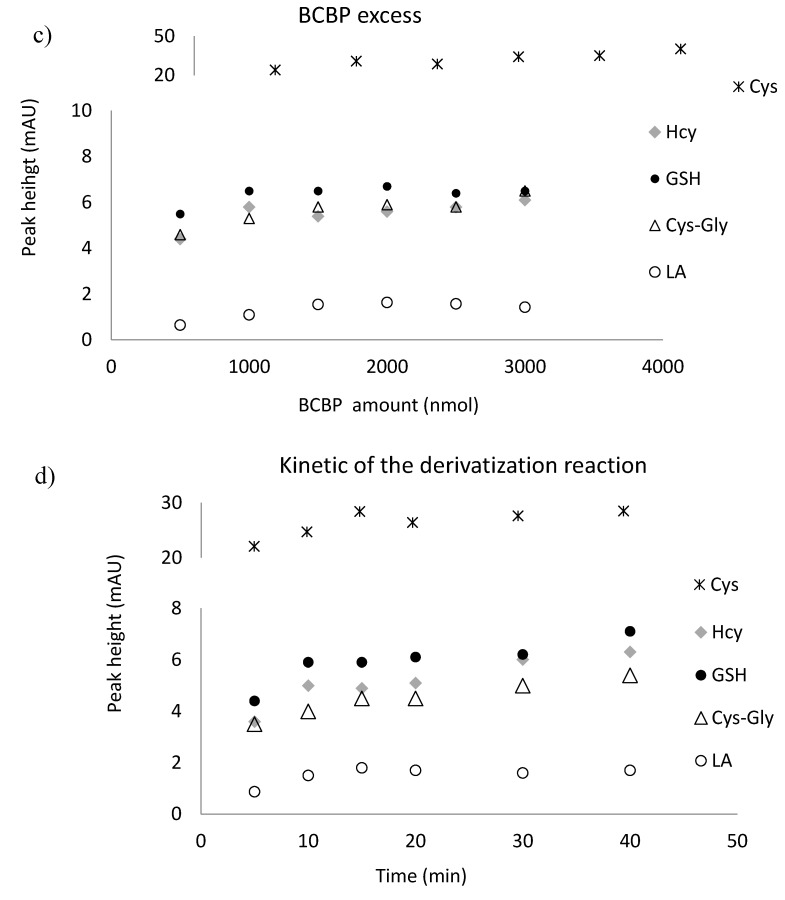
The influence of 1-benzyl-2-chloropyridinium bromide quantity, pH of the reaction mixture, and time on derivatization reaction yield of dihydrolipoic acid, reduced glutathione, cysteine, cysteinylglycine, and homocysteine. (**a**) Reaction of 1-benzyl-2-chloropyridinium bromide with dihydrolipoic acid carried out in 0.2 mol L^−1^, phosphate buffer, pH = 9.0; (**b**) reaction of 1-benzyl-2-chloropyridinium bromide with dihydrolipoic acid carried out in triple excess of the derivatizing reagent; (**c**) derivatization reaction carried out in 0.2 mol L^−1^, phosphate buffer, pH = 7.8; (**d**) derivatization reaction carried out in 0.2 mol L^−1^, phosphate buffer, pH = 7.8 in 2000-fold excess of the derivatizing reagent.

To continue studies on the proper parameters of LA and low-molecular-mass amino thiols, derivatization with BCPB was chosen. This compound reacts specifically with thiols via the –SH group to form stable 2-S-pyridinium derivatives with well-defined maximum absorption in the UV region (at 274 nm for the reagent and 321 nm for the derivative) and can be used in a wide range of pH [31,35]. In order to establish optimum conditions for the derivatization of LA and thiols, the pH of the buffer, the excess of BCPB, and time of derivatization reaction were tested. To estimate the stoichiometric molar ratio of the reagents, the continuous variation method for the reaction of LA with BCPB was applied. The derivatization reaction was carried out in 0.2 mol L^−1^ phosphate buffer, pH 6.0, and pH 9.0. The tests confirmed the presence of two different forms of LA derivatives and proved that these substrates react in the molar ratio of 1:1 (LA 1) and 1:2 (LA 2) (Figure 3). A similar relationship has not been mentioned in previously published studies. Additionally, we confirmed that the excess of BCPB (Figure 4a) and pH of the reaction mixture (Figure 4b) affect the derivatization efficiency and favor the reaction in which both –SH groups in the DHLA structure are blocked by BCPB. The high excess of the reagent positively affected the derivatization of all analytes of interest (Figure 4c). A complete derivatization reaction occurred after 10 min at pH 7.8 (Figure 4d).

### 2.2. Reduction of Disulfide Bonds

**Figure 5:**
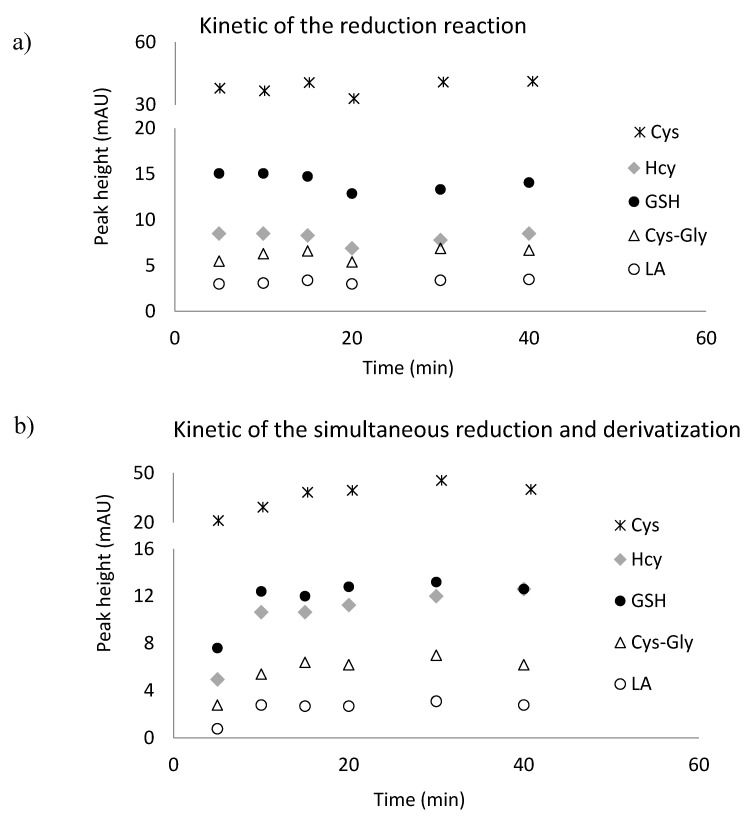
The influence of time on the reaction yield of *tris*(2-carboxyethyl)phosphine with disulfides and simultaneous reduction and derivatization reactions yields of *tris*(2-carboxyethyl)phosphine and 1-benzyl-2-chloropyridinium bromide with α-lipoic acid, oxidized glutathione, cystine, cysteinylglycine and homocystine. (**a**) Separate reactions of reduction and derivatization; (**b**) simultaneous reduction and derivatization reactions. Reactions carried out in 0.2 mol L^−1^, phosphate buffer, pH = 7.8.

LA and GSH, Cys, Hcy occur in the human body, mainly in the oxidative form. To release the thiol groups and make them available for the derivatizing reagent, the reduction reaction is required. For this purpose *tris*(2-carboxyethyl)phosphine (TCEP) solution was used. This reagent is known to be powerful also under mild conditions of pH and temperature [28,31,33]. In our approach, disulfide bonds were reduced in 10 min at room temperature at pH 7.8 (Figure 5a).

**Figure 6:**
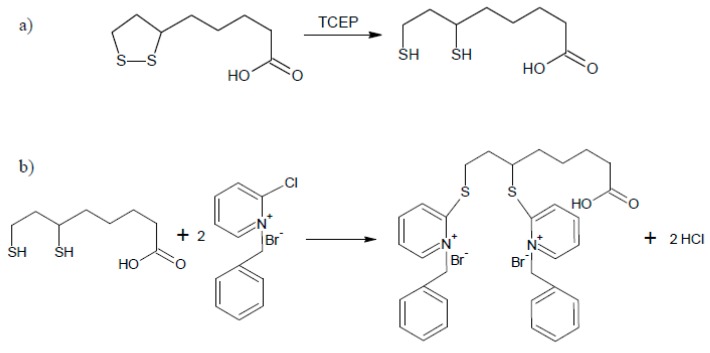
Schemes of chemical reduction reaction equation of α-lipoic acid with *tris*-(2-carboxyethyl)phosphine (**a**) and chemical derivatization reaction equation of dihydrolipoic acid with 1-benzyl-2-chloropyridinium bromide (**b**).

To reduce the number of steps and time of derivatization and reduction, we studied the efficiency of simultaneous reduction and derivatization reactions. For this experiment, the mixture 0.25 mol L^−1^ TCEP and 0.1 mol L^−1^ BCPB in 0.1 mol·L^−1^ NaOH was used. These reactions were completed at room temperature, pH 7.8 after 15 min (Figure 5b). This experiment confirmed the possibility of simultaneous reduction and derivatization of LA and low-molecular-mass amino thiols in plasma samples. The schemes of the reduction and derivatization reactions of LA have been presented in Figure 6.

### 2.3. LA and DHLA Separation from Proteins

LA possesses structural similarity to medium chain fatty acids, e.g., octanoic acid; for this reason, it is preferably bounded by sit II in HSA [36,37]. For an accurate separation of free LA, removal of the analyte from plasma proteins is essential to prevent underestimation. For removal of free LA from proteins, a different method based on liquid–liquid extraction [25,26,27,38], solid phase extraction [39], and deproteinization [26,38] was elaborated. For liquid–liquid extraction of LA from plasma samples, dichloromethane, diethyl ether, and ethyl acetate were considered. Results provided by other investigators indicated that the recovery of extraction of LA form proteins with the use of an acidic mixture of MeCN is 98% [21]. In our protocol to release free LA from proteins and to avoid an additional step of extraction and protein precipitation, 20 µL of acetonitrile and 1 mol L^−1^ HCl were added to the final mixture after derivatization. The addition of HCl was necessary to stop the simultaneous reduction and derivatization reactions, and improve the stability of the derivatives. The addition of MeCN is necessary to remove the LA–BCPB derivative from the protein surface.

### 2.4. Chromatographic Conditions

To avoid the step of protein precipitation, a chromatographic column dedicated to protein analysis was used for this assay. As was confirmed previously, the Aeris WIDPORE XB-C18 column can be successfully applied for this type of analysis [30]. Due to the unique separation parameters of the HPLC technique, it has been frequently used in the analyses of biological fluids. In spite of that, reversed-phase high performance liquid chromatography (RP-HPLC) has some limitations when samples containing a mix of compounds possessing different physicochemical properties, different size, and hydrophobicity of the particles are analyzed. To provide a good separation of the assay, the chromatographic conditions were optimized in terms of the content of MeCN and TCA in the mobile phase, pH of the mobile phase, gradient profile, and flow rate of the mobile phase to confirm that the method can efficiently separate all compounds of interest. The amount of the organic modifier and pH of the mobile phase were altered to affect changes in retention and selectivity, primarily by changing the hydrophobicity of the eluent and degree of ionization of the analytes.

**Figure 7:**
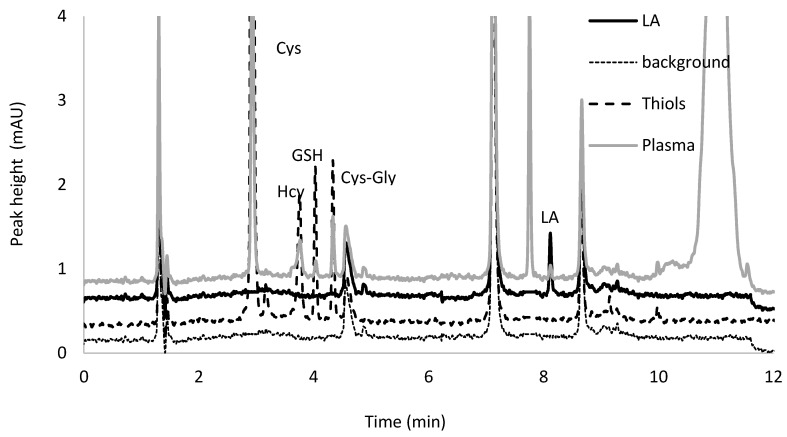
Typical chromatograms of background, pyridinium derivatives of α-lipoic acid, oxidized glutathione, cystine, cysteinylglycine and homocystine after simultaneous reduction and derivatization in proxy matrix spiked with analytes and plasma samples collected from an apparently healthy adult.

As is shown on the chromatogram depicted in Figure 7, the five BCPB derivatives of Hcy, Cys, GSH, Cys–Gly, and DHLA have been well separated. The pyridinium derivative of DHLA exhibits the highest hydrophobicity and elutes as the last. All analytes, including DHLA-BCPB, elute within 12 min in contrast to our earlier published protocols dedicated for low-molecular-mass thiols determination [40,41]. As can be seen in the chromatogram, optimization of the separation conditions lead to a very good resolution and good peak symmetry in all analytes of our interest.

### 2.5. Method Validation

#### 2.5.1. LOD and LOQ

According to the guidelines for biological sample analysis [42], limit of detection (LOD) and limit of quantification (LOQ) are defined as the lowest concentrations giving the signal/noise ratio of 3 and 10, respectively. The LODs for LA and low-molecular-mass thiols (Hcy, GSH, Cys, CysGly) in plasma samples were 0.08 and 0.12 nmol mL^−1^, respectively, while LOQs were 0.12 and 0.16 nmol mL^−1^, respectively.

#### 2.5.2. Linearity

For the plasma samples, six-point calibration plots were constructed for LA and low-molecular-mass thiols in triplicate. In both cases, the correlation coefficients were greater than 0.999. All calibration data, including regression equations, are shown in Table 1.

#### 2.5.3. Accuracy and Precision

The accuracy and precision of the proposed method were proven by adding known amounts of standard solutions of the analytes to plasma samples. Precision was calculated as the relative standard deviation, whereas accuracy was considered as the percentage of analyte recovery using the following formula:

accuracy (%) = 100% × (measured amount − endogenous content)/added amount.
(1)

Measured concentrations were assessed by the application of calibration curves obtained on that occasion. The estimated validation parameters were correct and met the requirements dedicated to biological sample analysis [42,43]. All detailed data are presented in Table 2.

### 2.6. Application to Authentic Plasma Samples

The analytical approach has been applied for the determination of Cys, Hcy, GSH, Cys–Gly, and LA in plasma samples collected from nine apparently healthy volunteers in the age range 27–49 years, supplemented with one dose of commercially available 100 mg LA capsules. In the analyzed samples the average content of the analytes of interest amounted to 130.3 ± 29.6 nmol mL^−1^ for Cys, 6.7 ± 2.4 nmol mL^−1^ for Hcy, 6.4 ± 4.5 nmol mL^−1^ for GSH, 15.5 ± 3.0 nmol mL^−1^ for Cys–Gly, and 5.0 ± 0.8 nmol mL^−1^ for LA. The results have confirmed the applicability of the elaborated assay for the analysis of human plasma samples.

## 3. Materials and Methods

### 3.1. Chemicals and Reagents

Oxidized glutathione, homocystine, cystine, cysteinylglycine, α-lipoic acid, and *tris*(2-carboxyethyl)phosphine were received from the Sigma Aldrich Company (St. Louis, MO, USA). The derivatization reagent, 1-benzyl-2-chloropyridinium bromide, was synthesized in our laboratory as described previously [44]. The HPLC gradient grade acetonitrile used for chromatographic analysis, hydrochloric acid (HCl) utilized for the standard solution preparation, sodium hydrogen phosphate heptahydrate (Na_2_HPO_4_·7H_2_O), sodium dihydrogen phosphate dihydrate (NaH_2_PO_4_·2H_2_O), sodium hydroxide and trichloroacetic acid were purchased from J.T. Baker (Deventer, The Netherlands). Deionized water was produced in our laboratory.

### 3.2. Instrumentation

All analyses were performed on a 1200 Series HPLC system (Agilent Technologies, Waldbronn, Germany) equipped with a quaternary pump, vacuum degasser, autosampler, module of temperature control, and spectrofluorometric detector. All analyses were controlled by HP ChemStation software. The Aeris WIDPORE XB-C18 (150 × 4.6 mm, 5 µm) column from Phenomenex, packed with 3.6 µm particles, was used for the analytes separation. Water used for the mobile phase preparation was distilled with the use of a Milli-QRG system from Millipore in Vienna, Austria. The pH of the phosphate buffer and mobile phases was controlled using a HI 221 pH meter, model Hanna Instruments, Woonsocket, RI, USA.

### 3.3. Human Plasma Samples

Samples collected from nine volunteers were studied. Volunteers were dosed with commercially available LA capsules (100 mg of LA). The supplement was provided in the morning, half an hour after breakfast. During the study, no additional medications were allowed except for LA. Blood was collected into vacutainer tubes containing EDTA by venipuncture, immediately placed on the ice, and centrifuged at 800× *g* for 15 min at room temperature. Plasma was used for the analyses without delay or stored at −80 °C.

All investigations were performed after approval by the Ethical Committee of the University of Lodz (decision identification code: 9/KBBN-UŁ/II/2017, approved on 6 November 2017). Informed consent forms were obtained from all volunteers involved in the project.

### 3.4. Stock Solutions

A stock solution of 0.1 mol L^−1^ LA was prepared in 1 mol L^−1^ NaOH. Stock solutions of 0.05 mol·L^−1^ Cys, Hcy, GSH, and Cys-Gly were prepared in 0.2 mol·L^−1^ HCl. All of them were kept at 4 °C for several days without a noticeable change in the analytes’ content. The working solutions were prepared by dilution with water as needed. Stock solution of 0.2 mol·L^−1^ BCBP and TCEP (0.125 mol·L^−1^) was prepared in 0.1 mol·L^−1^ NaOH. 0.2 mol L^−1^ pH 7.8 phosphate buffer was prepared freshly every day.

### 3.5. Human Plasma

A total of 50 µL of plasma was diluted with 150 µL of 0.2 mol L^−1^ pH 7.8 phosphate buffer and treated with 5 μL of mixture 0.2 mol L^−1^ BCBP and 0.125 mol L^−1^ TCEP for 15 min. In the next step, 30 µL of 1.0 mol L^−1^ HCl and 30 μL of MeCN was added. Of the final sample, 5 μL was injected into the chromatographic column.

### 3.6. HPLC conditions for Determination of LA and Low-Molecular-Mass Thiols in Human Plasma

The chromatographic separation of LA and low-molecular-mass thiols in human plasma was obtained in 12 min. The analytes were eluted by the mobile phase containing (A) 0.1% TCA adjusted to pH 2.25 with 1 mol L^−1^ NaOH and (B) acetonitrile with the gradient elution as follows: 0–5 min, 10–20% (B); 5–9 min, 20–45% (B), 9–11 min, 45–10% (B). For column equilibration, a 1 min post time was used. The flow rate of the mobile phase was 1 mL min^−1^. The peaks of 2-*S*-pyridinium derivatives of Cys, Hcy, GSH, Cys–Gly, and DHLA were monitored at 321 nm. All signals were identified by comparison of their retention times as well as diode-array spectra, taken at real-time of analysis, with that of the authentic standard. Separations were performed at room temperature.

### 3.7. Calibration and Validation Process

Calibration standards were prepared by spiking 50 µL of human plasma with appropriate disulfides to obtain the following concentrations: 0, 40, 100, 200, 300, 400 µmol L^−1^ plasma for Cys; 0, 2, 5, 10, 15, 20 µmol·L^−1^ for Hcy, GSH and Cys–Gly; and 0.0, 0.1, 1.0, 2.5, 4.0, 5.0 µmol L^−1^ for LA. Then the samples were processed according to the procedure in Section 3.5.

To investigate LOD and LOQ of the analytes of interest, a proxy matrix (0.9% NaCl in 10 mmol·L^−1^ phosphate buffer, pH 7.4) was spiked with decreasing concentrations of the standard solution of LA and low-molecular-mass thiols were subsequently subjected to all steps of the analytical procedure. The study was repeated until the signal-to-noise ratio reached 3:1 and 10:1 for LOD and LOQ, respectively.

## 4. Conclusions

In this paper, we propose a new method for the simultaneous separation and determination of Cys, Hcy, GSH Cys–Gly, and LA in human plasma. The assay is based on the simultaneous reduction with TCEP and derivatization with BCBP and elimination of the deproteinization step from the sample preparation protocol. Although in the literature, some assays dedicated to LA or GSH, or other amino thiols can be found, they do not allow the simultaneous determination of biologically important aminothiol antioxidants such as LA [24] and GSH [45] and other metabolically related low-molecular thiols [30]. The presented methodology exhibits some advantages when compared to other previously published reversed phase HPLC based methods. Our approach significantly simplifies and reduces the time taken by the sample preparation step. In this case, only simultaneous reduction of disulfide bonds and derivatization of thiols groups is involved. The step of deproteinization is eliminated. From an analytical point of view, our test is simple, fairly fast, sensitive, and does not require large sample volumes. Additionally, elimination of the deproteinization step allows us to prepare the samples in vials, which helps to reduce the number of polypropylene tubes and “plastic laboratory” waste. The validation parameters, including linearity, precision, and accuracy, were within the rules for biological samples.

The analytical approach has been successfully applied for the determination of Cys, Hcy, GSH, Cys–Gly, and LA in plasma samples collected from apparently healthy volunteers. The obtained results have confirmed the applicability of the elaborated assay for the analysis of human plasma samples. In our opinion, this method would act as a powerful analytical tool in high throughput screening of large numbers of samples. To the best of our knowledge, the proposed assay is the first that allows the simultaneous separation and determination of Cys, Hcy, GSH Cys–Gly, and LA in human plasma.

## Figures and Tables

**Table 1:** Validation data.

Analyte	Linear Range (nmol mL^−1^)	Regression Equation	R^2^	Imprecision (%)		Recovery (%)	
				Min.	Max.	Min.	Max.
Cys	40.0–400.0	y = 3.78x + 16.80	0.999	0.2	6.5	99.1	100.8
Hcy	2.0–20.0	y = 0.24x + 1.76	0.999	1.5	9.7	94	105.5
GSH	2.0–20.0	y = 0.24x + 0.87	0.998	1.2	8.4	98.4	106.2
Cys-Gly	2.0–20.0	y = 0.17x + 2.15	0.999	0.6	13.4	100.3	105.2
LA	0.12–5.0	y = 0.38x + 0.04	0.999	2.3	14.7	97.2	101.4

**Table 2:** Accuracy and precision.

Analyte	Concentration (nmol mL ^−1^)	Precision (%)		Accuracy (%)	
		Intra-day	Inter-day	Intra-day	Inter-day
Cys	40	11.3	3.3	100.9	113.4
	200	2.3	4.6	99.1	98.9
	400	0.5	0.5	95.3	93.8
Hcy	2	5.8	4.2	94	103
	10	5.8	0.5	105.5	100.5
	20	1.7	5.3	103	99.7
GSH	2	5.6	5.6	98.4	106.2
	10	4.2	2.9	104.1	102.2
	20	1.8	3.8	106.2	99
Cys–Gly	2	6.2	4	105.2	98.6
	10	4.2	1.1	103.6	100.3
	20	1.2	4	99.2	100.4
LA	0.12	4.1	9.5	101.4	101.1
	2.5	4	9.2	97.2	99.5
	5	3.6	3.7	101.1	100.2

## Abbreviations

BCPB	1-benzyl-2-chloropyridinium bromide
Cys	cysteine
Cys–Gly	cysteinylglycine
DHLA	dihydrolipoic acid
GSH	glutathione
LDL-C	low-density lipoprotein cholesterol
Hcy	homocysteine
HSA	human serum albumin
LA	α-lipoic acid
MeCN	acetonitrile
TCA	trichloroacetic acid
TCEP	*tris*(2-carboxyethyl)phosphine
